# The impact of parents' physical activity goals and parental attitudes on physical activity during leisure time among children in middle childhood

**DOI:** 10.3389/fpubh.2023.1170413

**Published:** 2023-07-03

**Authors:** Agata Kamionka, Małgorzata Lipowska, Sebastian Lizińczyk, Mariusz Lipowski

**Affiliations:** ^1^Faculty of Physical Culture, Gdansk University of Physical Education and Sport, Gdańsk, Poland; ^2^Faculty of Social Sciences, University of Gdansk, Gdańsk, Poland; ^3^Central Board of Prison Service, Ministry of Justice, Warsaw, Poland

**Keywords:** parent-child relationship, sport in leisure time, physical activity in families, parental attitudes, behavior modeling

## Abstract

**Introduction:**

Parents' supportiveness and health-promoting habits significantly affect the intensity of children's physical activity (PA) and the involvement of parents in their engagement in PA; in this domain, both the hours devoted to PA and PA goals can be assessed. The family plays an important role in shaping the physical and social organization of the environment for children aged 4–6 years.

**Methods:**

A total of 680 families with 5-year-old children (330 girls, 350 boys) took part in the study. Data were collected from these participants, who were recruited from preschools and primary schools in the Pomeranian region of Poland. The aim of this study was to determine whether the involvement of parents in PA mediates the influence of parental attitudes on the ways in which their children spend their leisure time.

**Results:**

The results showed that not all parental attitudes had direct impact on children's leisure time in PA and outside PA. Other aspects parental attitude had no significant impact on offspring's free time. Among fathers, only four aspects of parental attitude (namely, acceptance–rejection, inconsistency, autonomy, and overprotectiveness) had an impact on PA goals. Mothers' goals were influenced by the strength of a larger number of aspects of attitude (namely, acceptance–rejection, autonomy, inconsistency, over-demandingness, and overprotectiveness). Similarly, the strength of mothers' and fathers' acceptance–rejection attitudes, attitudes of autonomy, and overprotectiveness had an impact on their PA goals but were not directly linked to their children's leisure-time engagement in PA.

**Discussion:**

Not all parental attitudes have a direct impact on children's PA or non-PA leisure time. However, the goals of PA parents have been recognized influence the leisure time of children in PA and outside PA. The most statistically significant relationship for both mothers and fathers was between parental attitudes and PA goals. Parental attitudes do not play a significant role in explaining involvement in PA or lack of it in leisure time among 5-year-old children.

## 1. Introduction

Physical activity (PA), defined as any type of PA practiced at any skill level and for the enjoyment of the individual, is an important part of a healthy lifestyle for both adults (1–4) and children (5, 6). Research has shown that exercise has a positive influence on body weight (7, 8), mental health (9), cognitive functioning in children and in older adults (10–12), general self-esteem (13), and quality of PA in children (14). Additionally, the support of parents and their own health-promoting habits significantly affect the intensity and quality of PA undertaken within a family (15–17). The family plays an important role in shaping the physical and social organization of the environment for children aged 4–6 years (18, 19). Preschool (20, 21) and school (22–24) also significantly influence a child's relationship with exercise. Children's engagement in PA, especially that of children attending kindergarten, is largely dependent on adult caregivers who provide them with opportunities for PA (25).

The ways in which children spend their leisure time depend on their parents' involvement in PA and their health habits (17, 26). Parental behaviors in relation to their children's PA provide insight into how family lifestyles influence the effectiveness of family-based interventions. There is a clear, measurable relationship between parents' and children's PA and the quality of their leisure time while sedentary (27, 28). It is very difficult to find literature reporting on the ultimate effects of family-related factors on PA among 6-year-old children. However, it is known that a sedentary lifestyle among 5-year-old children leads to body weight issues and affects the quality of PA. Increasing numbers of children are obese or overweight, thereby reducing their PA (29). Epidemiological reports from Europe, the USA, and other parts of the world indicate that children are devoting less time to PA (30).

Regular exercise in childhood becomes a habit and translates into pro-health behaviors in adult life (31). A 2018 study of Polish children showed that only 20% of children meet the requirements for daily PA and participate in organized sports activities. It is concerning that only 50% of parents encourage their children to undertake such activities (32). In 2017, the intensity of physical exertion among the adult Polish population was found to be mostly low, at 56% (33). The European Obesity Monitoring Project (34) found that young children spend significant amounts of time in front of the TV and computer. Furthermore, 80% of children and adolescents between 5 and 17 years of age do not participate in sufficient PA. Data from the WHO suggest that one in four adults does not participate in sufficient PA.

Moreover, as countries grow economically, levels of inactivity among the population increase and can reach as high as 70%. Increased levels of physical inactivity have a negative impact on healthcare systems, the environment, economic development, community welfare, and quality of life (30). The relationship between physical activity and parental attitudes is an interesting and important one; exploring this connection can potentially lead to opportunities for improved education of parents and preventative measures related to the quality of PA during family leisure time.

An important way in which parents influence their children is through their attitudes, which can be understood as a cognitive–aspirational–affective framework acquired by parents that shapes their behavior toward their child (35). Researchers have developed various typologies of parental attitudes, with the terrestrial typology being the most well-known, as referenced by Plopa. This typology classifies parental attitudes into five distinct types: (1) acceptance–rejection: under acceptance, a parent unconditionally accepts the child as they are, teaches the child acceptance of others, and instills in them a sense of trust for the world and people, while under a parental attitude of rejection, interaction with the child does not give the parent pleasure and satisfaction; (2) overly demanding attitude: the parent demands absolute obedience and submission from the child, not relenting in the face of opposition and criticism, and accepts only those of the child's plans, actions, and aspirations that are consistent with the parent's views, expectations, and ideas; (3) attitude of autonomy: the parent understands the child's growing need for independence and development, and treats the child sensitively and in accordance with their age and abilities; (4) inconsistent attitude: the parent's attitude toward their child is unstable, depending on their own mood or well-being, which seeps into interpersonal relationships in the family; and (5) overprotective attitude: the parent, guided by their belief in love for their child, cares too much about their child and wants to know about everything that concerns them (36).

The family as a system creates an internal environment that shapes parental behavior and the functioning of the child (37). In the literature, the sex of the parent is reported to be a significant variable that influences the parental attitudes presented to children. Mothers and fathers may present similar or different attitudes toward their children in different areas of their lives. It has been observed that, when preparing a child for school, most mothers adopt a democratic attitude, followed by an overprotective attitude, then a liberal attitude, and finally, an authoritarian attitude. Fathers present a similar pattern in the attitudes they adopt toward their children (38). Lipowska et al. (39) demonstrated that both mothers and fathers are most characterized by inconsistent and demanding attitudes and least by overprotective attitudes and attitudes of autonomy. Mothers exhibit acceptance–rejection attitudes, demanding attitudes, and attitudes of autonomy at higher rates than fathers (39). There is a slightly significant relationship between authoritarian parenting attitudes and children's play behaviors and behaviors related to social competencies (40). Parental attitudes affect various spheres of a child's life, in particular the types of activities undertaken, and are important for the education that occurs within the family (37).

### 1.1. Goals and hypotheses of this study

The aim of this study was to determine whether the involvement of parents in PA mediates the influence of parental attitudes on the ways in which their children spend their leisure time. To this end, we formulated two hypotheses:

Hypothesis 1. Parental attitudes impact how much of their leisure time children spend engaging in PA.
Hypothesis 2. Parents' own PA goals and involvement in PA mediate the relationship between parental attitudes and how their children spend their leisure time.

**Figure 1:**
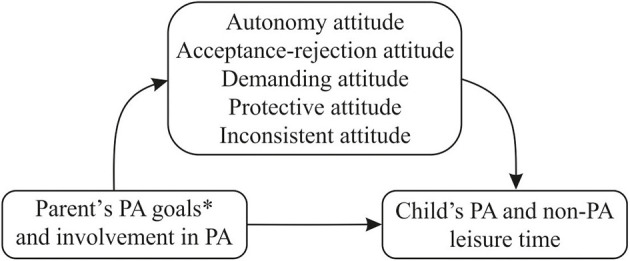
A mediation model for the parent–child dyad. *Parents' goals: (1) health (right levels of: blood pressure, cholesterol, body mass, etc.); (2) physical fitness, being “in shape”; (3) company of other people (4); fit, shapely body (beauty, sculpted and firm body); (5) well-being; (6) being physically active and fit according to fashion; (7) boosting confidence, gaining appreciation from others; (8) pleasure from physical activity; (9) escape from everyday life; (10) managing stress; (11) fulfilling the need for activity; and (12) promoting physical activity by setting a behavior example.

We created a mediation model for dyads of parents and children, representing these hypotheses about the relationships between the variables (Figure 1).

## 2. Materials and methods

### 2.1. Participants

**Figure 2:**
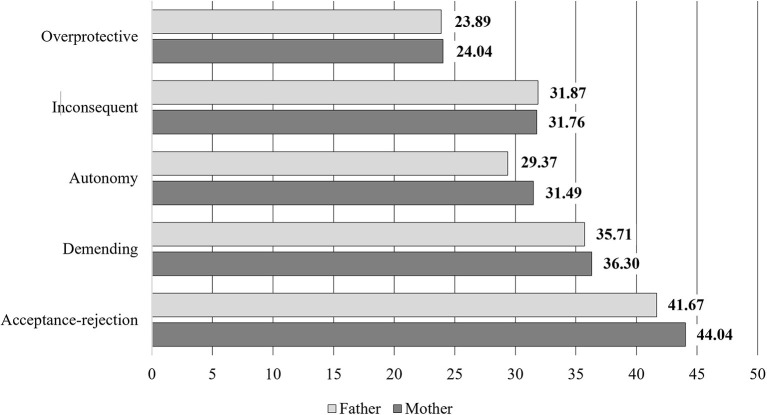
Descriptive statistics on parental values and attitudes.

A total of 680 families with 5-year-old children (330 girls and 350 boys; M_age_ = 5.7, SD = 0.32) took part in the study. All children attended educational institutions in the Pomerania region of northern Poland. To analyze the specific relationships between parents and children in terms of involvement in PA, only mother–father–child triads were invited to participate in the study. The researchers chose this age because children's motor skills undergo significant changes at this stage of development (41). Before the age of 5, children acquire fluid body movements and balance, and learn to alternate their movements. Subsequently, with each passing year, the child's movements become more automatic and undergo improvement; in the early school period, motor development has reached a sufficient level to constitute the foundation for learning other activities (42). In addition, starting school in the “zero” grade is associated with the child's first partially independent decisions to engage in health-promoting or health-threatening behaviors. The ages of 5–6 years therefore constitutes an ideal period to investigate how parental attitudes and PA goals affect engagement in PA, or lack thereof, by children during their leisure time in middle childhood. Figure 2 presents descriptive statistics (mean values and standard deviation) on the results of the parenting attitudes presented by mothers and fathers.

**Figure 3:**
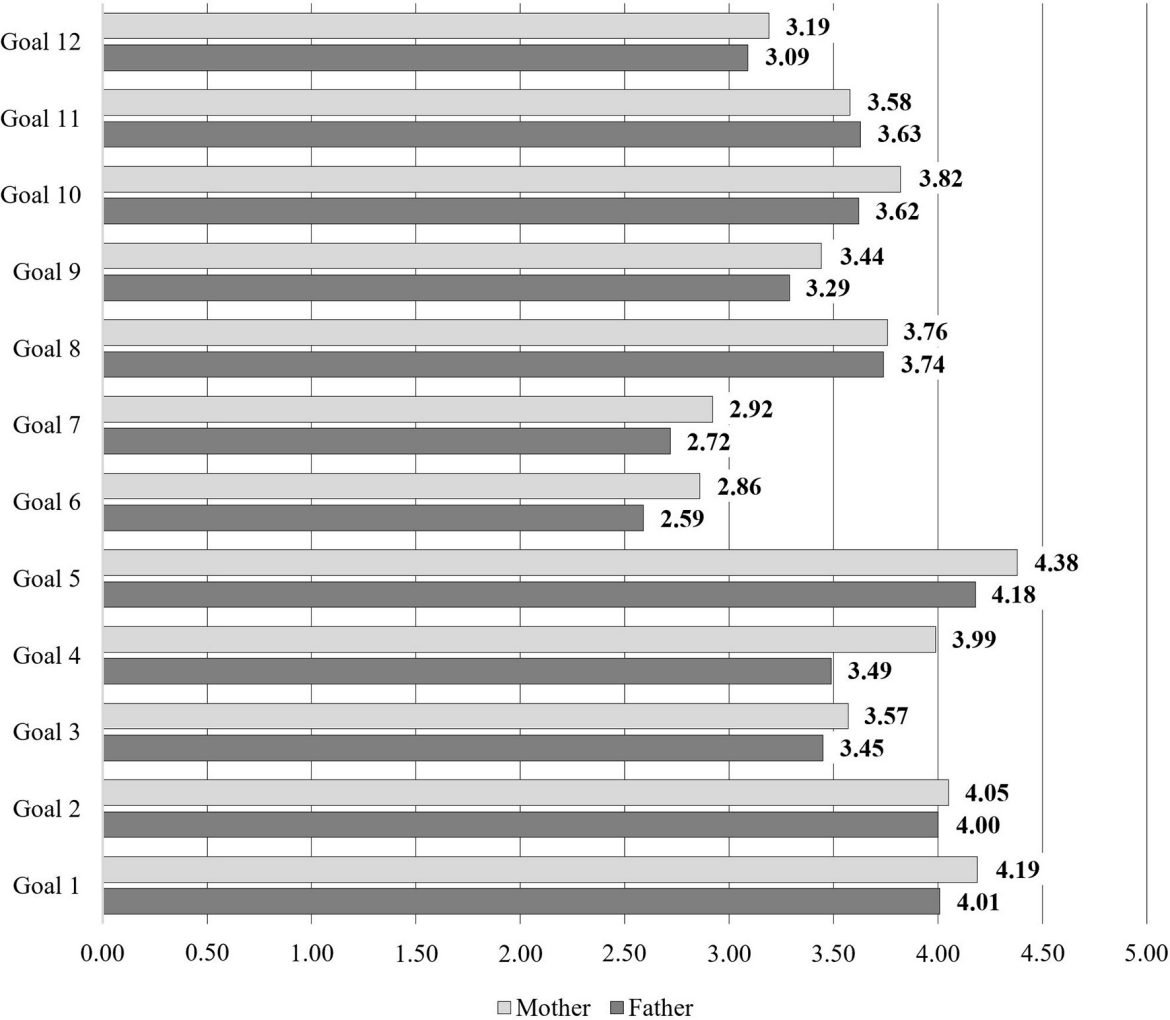
Descriptive statistics on the values and goals underlying parents' engagement in PA. Goal 1: health (right levels of: blood pressure, cholesterol, body mass, etc.); Goal 2: physical fitness, being “in shape”; Goal 3: company of other people; Goal 4: fit, shapely body (beauty, sculpted and firm body); Goal 5: well-being; Goal 6: being physically active and fit according to fashion; Goal 7: boosting confidence, gaining appreciation from others; Goal 8: pleasure from physical activity; Goal 9: escape from everyday life; Goal 10: managing stress; Goal 11: fulfilling the need for activity; Goal 12: promoting physical activity by setting a behavior example.

Descriptive statistics on the goals of PA undertaken by mothers and fathers are presented in Figure 3.

Table 1 presents the mean, median, and standard deviation for parental attitudes and PA goals.

**Table 1:** Mean, median, and standard deviation for each variable relating to parental attitudes and PA goals.

**Mothers**	***M***	***Me***	**Min**	**Max**	***SD***
SPR: acceptance–rejection	44.04	45.00	15.00	50.00	4.96
SPR: overly demanding	36.30	36.00	22.00	50.00	4.48
SPR: autonomy	31.49	31.00	12.00	50.00	7.50
SPR: inconsistent	31.76	32.00	13.00	48.00	6.40
SPR: overprotective	24.04	24.00	10.00	44.00	6.97
Goal 1: health (right levels of: blood pressure, cholesterol, body mass, etc.)	4.19	4.00	1.00	5.00	0.92
Goal 2: physical fitness, being “in shape”	4.05	4.00	1.00	5.00	0.88
Goal 3: company of other people	3.57	4.00	1.00	5.00	1.15
Goal 4: fit, shapely body (beauty, sculpted and firm body)	3.99	4.00	0.00	5.00	0.95
Goal 5: well-being	4.38	5.00	1.00	5.00	0.80
Goal 6: being physically active and fit according to fashion	2.86	3.00	0.00	5.00	1.23
Goal 7: boosting confidence, gaining appreciation from others	2.92	3.00	1.00	5.00	1.21
Goal 8: pleasure from physical activity	3.76	4.00	1.00	5.00	1.01
Goal 9: escape from everyday life	3.44	4.00	1.00	5.00	1.12
Goal 10: managing stress	3.82	4.00	1.00	6.00	1.07
Goal 11: fulfilling the need for activity	3.58	4.00	1.00	5.00	1.06
Goal 12: promoting physical activity by setting a behavior example	3.19	3.00	0.00	5.00	1.17
**Fathers**					
SPR: acceptance–rejection	41.67	42.00	16.00	50.00	6.20
SPR: overly demanding	35.71	36.00	19.00	49.00	4.77
SPR: autonomy	29.37	30.00	10.00	50.00	7.76
SPR: inconsistent	31.87	33.00	13.00	49.00	6.97
SPR: overprotective	23.89	24.00	10.00	44.00	7.85
Goal 1: health (right levels of: blood pressure, cholesterol, body mass, etc.)	4.01	4.00	1.00	5.00	1.02
Goal 2: physical fitness, being “in shape”	4.00	4.00	1.00	5.00	0.96
Goal 3: company of other people	3.45	4.00	1.00	5.00	1.15
Goal 4: fit, shapely body (beauty, sculpted and firm body)	3.49	4.00	0.00	5.00	1.09
Goal 5: well-being	4.18	4.00	1.00	5.00	0.88
Goal 6: being physically active and fit according to fashion	2.59	3.00	0.00	5.00	1.20
Goal 7: boosting confidence, gaining appreciation from others	2.72	3.00	1.00	5.00	1.20
Goal 8: pleasure from physical activity	3.74	4.00	0.00	5.00	1.10
Goal 9: escape from everyday life	3.29	3.00	0.00	5.00	1.19
Goal 10: managing stress	3.62	4.00	0.00	5.00	1.12
Goal 11: fulfilling the need for activity	3.63	4.00	0.00	5.00	1.08
Goal 12: promoting physical activity by setting a behavior example	3.09	3.00	0.00	5.00	1.21

M, mean; Me, median; Min, minimum; Max, maximum; SD, standard deviation; SPR, Parental Attitudes Scale.

### 2.2. Procedure

Data were collected from participants recruited from preschools and primary schools in the Pomeranian region of Poland. The research was conducted in 2017–2019. Children were assessed individually at educational centers; the data used for this study were part of a larger project, and the detailed recruitment procedure is described elsewhere (43). Prior to the study, written informed consent was obtained from all parents/carers, who were also informed that they could discontinue the participation of their children at any time without repercussions.

This study was conducted in accordance with the Code of Ethics of the World Medical Association (Declaration of Helsinki) for experiments involving collection of data from humans. The protocol of this study was approved by the Ethics Board for Research Projects at the Institute of Psychology, University of Gdansk, Poland (decision no. 17/2013).

### 2.3. Research tools

We collected several different kinds of information using the Parental Attitudes Scale (SPR), the Inventory of Physical Activity Objectives (IPAO), and the Survey for the Assessment of Children's PA. The authors conducted interviews of the parents, which included questions about the type and amount of time spent by children and parents on PA outside their home and educational institution. The data used for this study were part of a larger survey, and the questionnaires that formed this study took approximately 20 min to complete.

#### 2.3.1. The parental attitudes scale (SPR)

The questionnaire consists of 50 diagnostic statements grouped into five dimensions corresponding to the five different parental attitudes: acceptance–rejection, autonomy, overprotective, overly demanding, and inconsistent. The “acceptance–rejection” category describes the level of parental acceptance of the child, where low scores indicate distant, insensitive, and rejecting attitudes (psychologically and physically) toward the child, and high scores point to accepting, supportive, and sensitive parental attitudes. The “autonomy” category measures the level of parental respect for the child's needs and the parent's ability to adjust their behavior to the child's developmental needs (the higher the score, the higher the acceptance of the child's autonomy). The “overprotective” category measures the tendency to consider one's own child vulnerable, helpless, and dependent; the higher the score on this dimension, the higher the intensity of a distrustful attitude and preoccupation with the child's future. The “demanding” category focuses on parental expectations of the child; high scores in this category are associated with more rigid and critical attitudes toward the child and valuing of submissive behavior. Finally, the “inconsistent” dimension measures parental inclination toward inconsistent reactions dependent on their mood and daily situation, and general parental emotional instability (36).

The respondents were asked to rate on a five-item scale how strongly they agreed or disagreed with statements corresponding to each of these five dimensions of parental attitudes. The questionnaire was administered using two different versions to examine maternal and paternal attitudes. The Cronbach's alpha reliability coefficients for our sample were 0.85 for mothers and 0.86 for fathers.

#### 2.3.2. The inventory of PA objectives

The Inventory of PA Objectives (IPAO) by Lipowski and Zaleski (44) was used to collect detailed interviews regarding the athletic history of the parents and their current engagement in various forms of PA. The questionnaire contains questions regarding whether the respondent has participated in sports in the past, what kind of sports they practiced, for how long, and at what level. Analysis of the number of hours per month currently devoted to certain types of PA (e.g., gym, swimming, running, team sports, martial arts) is an important part of the questionnaire; additionally, respondents indicated whether they engaged in these activities regularly or sporadically. The questionnaire also allows for the analysis of the goals with which respondents undertake PA (44). However, this aspect was not used in the current study. The Cronbach's reliability coefficient for the IPAO was 0.79.

#### 2.3.3. The survey for the assessment of children's PA

The Survey for the Assessment of Children's PA was developed by (43) for the current project. Parents answered questions indicating the number of hours per week that their child devoted to outdoor play, participation in organized sports classes, walks with parents, riding a bicycle, and playing at playgrounds. Parents were also asked to list additional school classes (outside school/preschool) in which their child participated and to provide an assessment the number of hours per week their child devoted to these classes. The survey also contains questions regarding the types of sports equipment owned by the child. Finally, parents were asked to assess their children's physical fitness on a scale from 1 (“my child has very low levels of physical fitness”) to 5 (“my child has very high levels of physical fitness”).

## 3. Results and statistical analysis

In the course of further analysis, a number of statistical calculations were conducted (analysis of the normality of the distribution and correlations between the variables) to assess the suitability of the data for verification of the proposed model. As the data were suitable, the model was tested in relation to the raw data collected. The variables were standardized, and a path model was subsequently developed based on the theoretical model presented above (Figure 1). The AMOS 21 software tool, which is part of the SPSS program, was used to conduct this analysis.

Due to the large number of interactions tested, we report only the final outcomes of the path analysis in this article, presenting only significant path coefficients. Proxy variables and independent variables were introduced gradually to achieve the best possible adaptation of the path model to the raw data.

The data were statistically analyzed using the Statistica program, and path models were constructed for parent–child dyads using AMOSS SPSS v.18. The path models obtained for mother–child and father–child dyads are presented in Tables 1, 2, respectively. Under the theoretical models for both father–child and mother–child dyads, it was assumed that, beyond the parent's PA goals, the number of hours spent per month on PA by the parent would also be of some importance. However, the number of hours spent per month on PA by the mother or father had no statistically significant impact on the amount of the child's leisure time spent engaging or not engaging in PA, and no correlation was found between parental attitudes and PA goals.

**Table 2:** The obtained path model for the mother–child dyad.

**Path model: MOTHER**			**Path factor**	***p***
Goal 4: fit, shapely body	< –	SPR_M_acceptance–rejection	0.120	^*^
Goal 5: well-being	< –	SPR_M_ acceptance–rejection	0.162	^**^
Goal 6: being active	< –	SPR_M_ acceptance–rejection	−0.127	^*^
Goal 7: confidence and appreciation	< –	SPR_M_ acceptance–rejection	−0.297	^***^
Goal 10: managing stress	< –	SPR_M_ acceptance–rejection	0.138	^*^
Goal 3: company of other people	< –	SPR_M_ autonomy	0.260	^***^
Goal 6: being active	< –	SPR_M_ autonomy	0.259	^***^
Goal 7: confidence and appreciation	< –	SPR_M_ autonomy	0.347	^***^
Goal 9: escape from everyday life	< –	SPR_M_ autonomy	0.206	^***^
PA leisure time without the child	< –	SPR_M_ autonomy	0.171	^**^
Goal 3: company of other people	< –	SPR_M_inconsistent	−0.124	^*^
Goal 4: fit, shapely body	< –	SPR_M_ inconsistent	−0.174	^**^
Goal 3: company of other people	< –	SPR_M_overprotective	−0.157	^**^
Goal 7: confidence and appreciation	< –	SPR_M_overprotective	−0.142	^*^
Goal 10: managing stress	< –	SPR_M_ overprotective	0.123	^*^
Goal 8: pleasure from PA	< –	SPR_M_over-demanding	0.183	^***^
Goal 9: escape from everyday life	< –	SPR_M_ over-demanding.	0.129	^**^
Goal 10: managing stress	< –	SPR_M_ over-demanding	0.125	^**^
Goal 11: fulfilling the need for activity	< –	SPR_M_ over-demanding	0.173	^***^
Goal 12: promoting PA	< –	SPR_M_ over-demanding	0.136	^**^
Efficiency assessment	< –	SPR_M_ over-demanding	0.179	^***^
Assessment of child's fitness	< –	Goal 3: company of other people	0.091	^*^
Child's PA leisure time	< –	Goal 4: fit, shapely body	−0.084	^*^
PA leisure time without the child	< –	Goal 4: fit, shapely body	−0.112	^**^
PA leisure time without the child	< –	Goal 6: being active	0.101	^*^
Child's PA leisure time	< –	Goal 12: promoting PA	0.088	^*^
Assessment of child's fitness	< –	SPR_M_ over-demanding	0.179	^***^
PA leisure time without the child	< –	SPR_M_autonomy	0.171	^**^
Assessment of child's fitness	< –	Goal 3: company of other people	0.091	^*^
Child's PA leisure time	< –	Goal 4: fit, shapely body	−0.084	^*^
PA leisure time without the child	< –	Goal 4: fit, shapely body	−0.112	^**^
PA leisure time without the child	< –	Goal 6: being active	0.101	^*^
Child's PA leisure time	< –	Goal 12: promoting PA	0.088	^*^

^*^p < 0.05; ^**^p < 0.01; ^***^p < 0.001.

Indicators of fit: chi-squared test = 3.59, df = 1, p > 0.05; CFI = 0.999; RMSEA = 0.037.

### 3.1. Path mediation model for the mother–child dyad

The path model analysis for mothers showed that only parental attitude of autonomy and demanding parental attitude influenced mothers' assessments of their child's fitness and their non-PA leisure time. Parental attitudes had a significant impact on the mother's PA goals. Finally, the mother's PA goals had a direct impact on the child's leisure time spent engaging in PA and their fitness (Table 2).

#### 3.1.1. Attitude of autonomy

A strong attitude of autonomy toward one's child (being accepting and allowing freedom in decision-making) contributed to the mother's PA goal of spending time in other people's company and to her willingness to be physically active. Additionally, it can be observed that a strong attitude of autonomy increased the strength of the PA goals of boosting confidence and finding escape from everyday life. The mother's attitude of autonomy was statistically significant (*p* < 0.001) in its relationship with the aforementioned goals.

#### 3.1.2. Acceptance–rejection attitude

The greater the extent to which mothers showed acceptance toward their children, the less they needed to achieve the goal of boosting confidence. This relationship was statistically significant (*p* < 0.001). Additionally, it can be observed that a high degree of acceptance of the child was linked to the goal of well-being (*p* < 0.01). The strength of the goals of achieving a shapely body and managing stress increased with the mother's attitude of autonomy toward their child (*p* < 0.05).

#### 3.1.3. Demanding attitude

A demanding attitude in the mother increased the strength of the goals of seeking pleasure from PA and fulfilling the need for exercise (*p* < 0.001). An excessively demanding attitude was linked to the strength of the goals of escaping from everyday life, managing stress relief, and promoting PA by setting an example (*p* < 0.01).

#### 3.1.4. Protective attitude

A protective attitude in the mother reduced the strength of the goal of spending time in other people's company (*p* < 0.01). Additionally, higher levels of protectiveness were linked with lower strength of the goals of boosting confidence and managing stress (*p* < 0.05).

#### 3.1.5. Inconsistent attitude

A more inconsistent attitude was linked with higher strength of the goal of spending time in other people's company and an increased need for a fit, shapely body. The influence of an inconsistent attitude was the least statistically significant (*p* < 0.05).

#### 3.1.6. The influence of PA goals and parental attitudes on children's leisure activities in middle childhood

**Figure 4:**
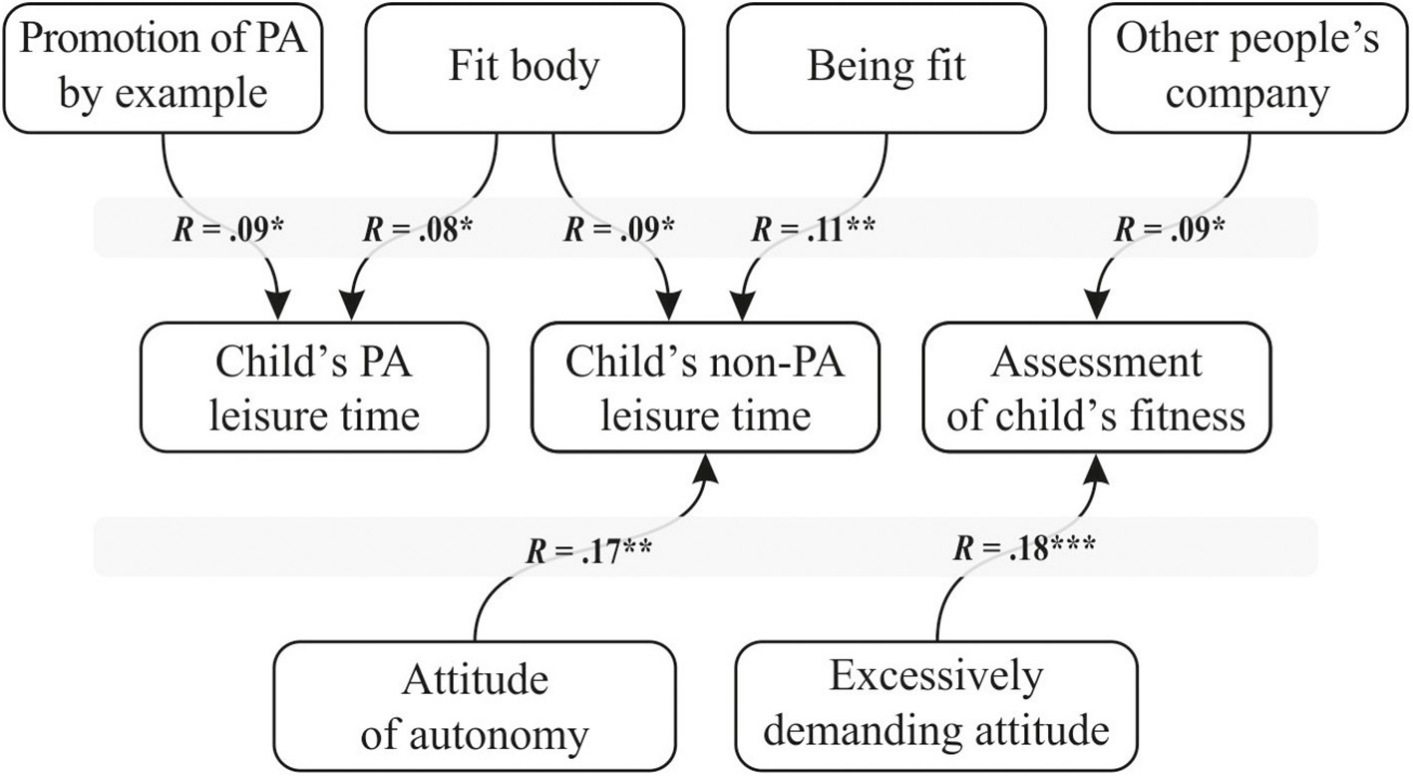
The obtained path model of the relationships between the mother's PA goals and parental attitudes and the child's engagement in PA. ^*^p < 0.05; ^**^p < 0.01; ^***^p < 0.001.

Mothers' attitudes of autonomy toward their children was linked with more non-PA leisure time among children (*p* < 0.01). However, a demanding attitude was important in mothers' assessments of their children's physical fitness: the more demanding the mother's attitude, the higher her assessment of the child's fitness (*p* < 0.001). A mother's PA goals had a greater influence on the child's engagement in PA, or lack thereof. The time spent by the child on non-PA activities outside preschool and the child's leisure time spent on PA both decreased as the strength of the mother's desire for a shapely body increased. The strength of the goal of being active increased the amount of leisure time spent on non-PA activities (*p* < 0.05). Finally, the strength of the goal of promoting PA by setting an example increased the child's PA time outside preschool (*p* < 0.05). Overall, parental attitudes had little effect on the dependent variables (Figure 4).

### 3.2. Path mediation model for the father–child dyad

The path model analysis for the father–child dyad showed that only the degree of inconsistent attitude in the fathers influenced the child's non-PA leisure time. Additionally, the strength of inconsistent attitude influenced the father's assessment of the child's fitness. Fathers' parental attitudes had little influence on the child's PA and non-PA leisure time. However, fathers' parental attitudes were linked to their own PA goals, and these goals had a direct impact on their assessment of the child's fitness and on the child's PA and non-PA leisure time (Table 3).

**Table 3:** The obtained path model for the father–child dyad.

**Path model: FATHER**			**Path factor**	***p***
Goal 5: well-being	< –	SPR_O_ acceptance–rejection	0.229	^***^
Goal 8: pleasure from PA	< –	SPR_O_ acceptance–rejection	0.259	^***^
Goal 10: managing stress	< –	SPR_O_ acceptance–rejection	0.154	^*^
Goal 11: fulfilling the need for activity	< –	SPR_O_ acceptance–rejection	0.241	^***^
Goal 12: promoting PA	< –	SPR_O_ acceptance–rejection	0.179	^**^
Goal 3: company of other people	< –	SPR_O_autonomy	0.212	^***^
Goal 6: being active	< –	SPR_O_autonomy	0.194	^**^
Goal 7: confidence and appreciation	< –	SPR_O_autonomy	0.212	^***^
Goal 4: fit, shapely body	< –	SPR_O_ inconsistent	0.131	^*^
PA leisure time without the child	< –	SPR_O_ inconsistent	0.149	^*^
Assessment of child's fitness	< –	SPR_O_ inconsistent	−0.142	^*^
Goal 5: well-being	< –	SPR_O_ overprotective	−0.181	^**^
Goal 6: being active	< –	SPR_O_ overprotective	−0.129	^*^
Child's PA leisure time	< –	Goal 4: fit, shapely body	−0.090	^*^
PA leisure time without the child	< –	Goal 4: fit, shapely body	−0.091	^*^
Assessment of child's fitness	< –	Goal 4: fit, shapely body	−0.113	^**^
Child's PA leisure time	< –	Goal 5: well-being	0.096	^*^
Child's PA leisure time	< –	Goal 8: pleasure from PA	−0.117	^**^
PA leisure time without the child	< –	Goal 11: fulfilling the need for activity	−0.149	^**^
PA leisure time without the child	< –	SPR_O_ inconsistent	0.149	^*^
Assessment of child's fitness	< –	SPR_O_ inconsistent	−0.142	^*^
Child's PA leisure time	< –	Goal 4: fit, shapely body	−0.090	^*^
PA leisure time without the child	< –	Goal 4: fit, shapely body	−0.091	^*^
Assessment of child's fitness	< –	Goal 4: fit, shapely body	−0.113	^**^
Child's PA leisure time	< –	Goal 5: well-being	0.096	^*^
Child's PA leisure time	< –	Goal 8: pleasure from PA	−0.117	^**^
PA leisure time without the child	< –	Goal 11: fulfilling the need for activity	−0.149	^**^

^*^p < 0.05; ^**^p < 0.01; ^**^p < 0.001.

Indicators of fit: chi-squared test = 1.19, df = 1, p > 0.05; CFI = 0.999; RMSEA = 0.012.

#### 3.2.1. Attitude of autonomy

The stronger a father's attitude of autonomy toward their child, the greater the strength of his goals to spend time in other people's company during PA (*p* < 0.001) and to be active. Additionally, the stronger the father's attitude of autonomy, the greater the strength of his goal of boosting confidence (*p* < 0.001).

#### 3.2.2. Acceptance–rejection attitude

The stronger a father's attitude of acceptance, the greater the strength of his goals of fulfilling the need for exercise, well-being, and pleasure (*p* < 0.001). Acceptance also increased with the strength of the goal of promoting PA by setting an example (*p* < 0.01). Lower levels of acceptance toward the child increased the fathers' stress relief through participation in PA (*p* < 0.05).

#### 3.2.3. Inconsistent attitude

A low level of inconsistent attitude increased the child's amount of PA leisure time and influenced the strength of the father's goal of achieving a fit, shapely body (*p* < 0.05). Additionally, the lower the father's level of inconsistency, the lower his assessment of the child's fitness (*p* < 0.05).

#### 3.2.4. Demanding and protective attitudes

The less demanding a father's attitude, the less emphasis he placed on the goal of being physically active (*p* < 0.05). A more protective attitude led to a decrease in the strength of the goal of promoting well-being (*p* < 0.01).

#### 3.2.5. The influence of PA goals and parental attitudes on children's leisure activities in middle childhood

**Figure 5:**
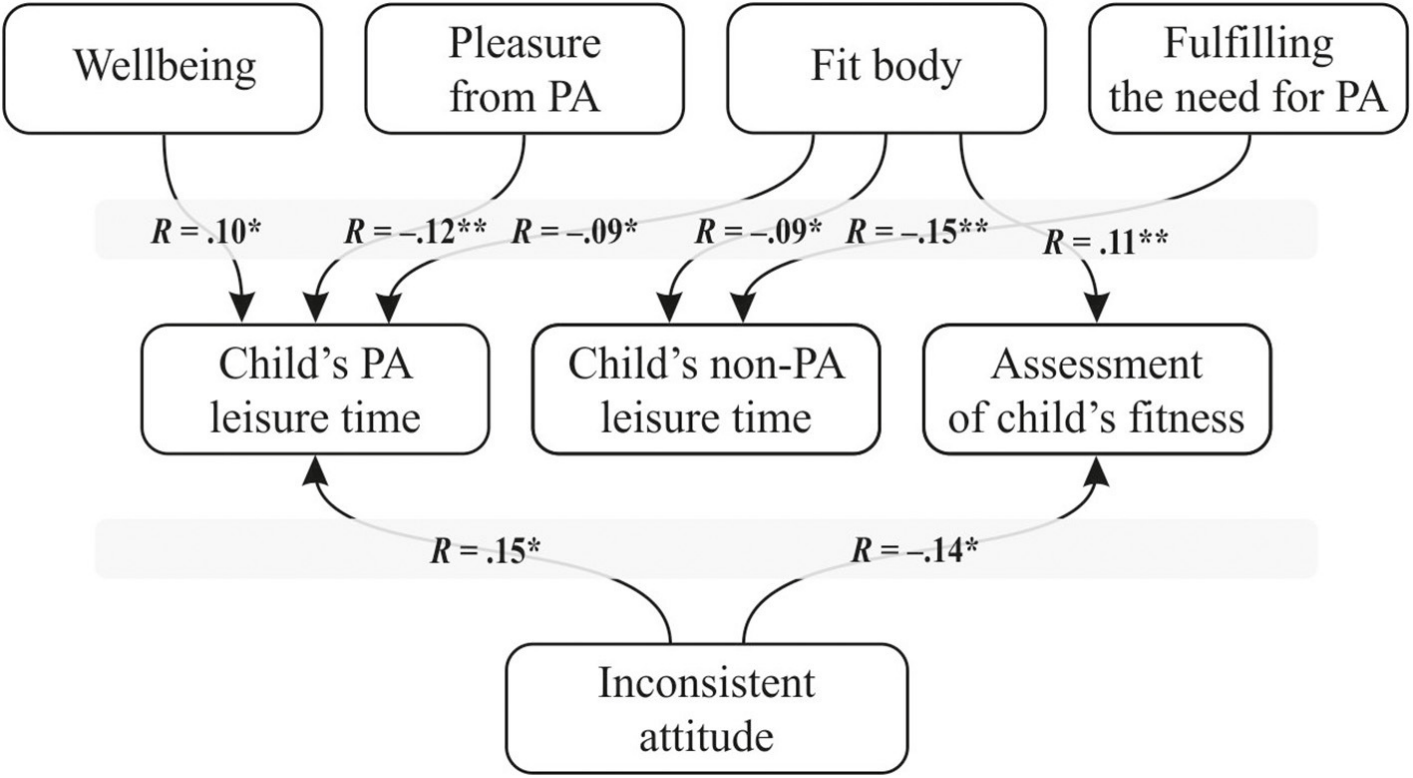
The obtained path model of the relationships between the father's PA goals and parental attitudes and the child's engagement in PA. ^*^p < 0.05; ^**^p < 0.01.

Fathers' attitudes had little effect on the dependent variables. Only the strength of inconsistency in attitude influenced the child's PA leisure time and the father's assessment of the child's fitness. Fathers' PA goals were of greater importance in their children's leisure time. The lower the father's desire to achieve a fit, shapely body, the less of the child's leisure time was devoted to PA (*p* < 0.05). The greater the father's desire for a fit, shapely body, the lower his assessment of the child's fitness (*p* < 0.01). The goal of well-being was linked to increased leisure time spent engaging in PA (*p* < 0.05). A strong need for PA in fathers was linked to less non-PA leisure time (*p* < 0.01). Finally, the strength of fathers' pleasure from PA was also linked to less PA leisure time (Figure 5).

### 3.3. Comparison of the obtained mother–child and father–child dyad models

In fathers, only four types of attitudes had an impact on PA goals: acceptance–rejection, inconsistency, autonomy, and overprotectiveness. Mothers' goals were influenced by a larger number of attitudes, acceptance–rejection, autonomy, inconsistency, over-demandingness, and overprotectiveness. Similarly, mothers' and fathers' attitudes of acceptance–rejection, autonomy, and overprotectiveness had an impact on their own PA goals, but were not directly linked to their children's PA leisure time. The similarity of the attitudes exhibited may be related to parents' shared beliefs regarding the upbringing of their children. In addition, these attitudes may be related to the child's physical and mental development via their influence on the parents' PA goals. In the future, this research model should be expanded to include other variables in order to define more precisely how parental attitudes affect PA in children in middle childhood. The models showed that the goal of achieving a fit, shapely body is important for parents of both sexes and affects the amount of leisure time the child spends on PA and non-PA activities. For fathers, other goals identified as influencing the child's PA were pleasure, satisfying the need for activity, and well-being, while in the case of the mother, it was important to be physically active and to promote PA by setting an example. The parents' assessment of the child's fitness depended on the degree of demandingness of the father's attitude and the strength of the goal of spending time in the company of other people. For fathers, the assessment of the child's fitness depended on the degree of inconsistency of his attitude and his desire to achieve a fit, shapely body.

## 4. Discussion

In this study, neither the parental attitudes of the mother nor those of the father had a direct impact on the amount of leisure time that their children spent on PA. However, parents' attitudes were directly related to the mothers' and fathers' goals for PA, and these PA goals did have a direct impact on their children's PA leisure time. In fathers, only four types of attitude had an impact on their PA-related goals: acceptance–rejection, inconsistency, autonomy, and overprotectiveness. A larger number of attitudes were relevant among mothers: acceptance–rejection, autonomy, inconsistency, over-demandingness, and overprotectiveness. The child's gender was not a significant differentiating factor, and both parents displayed similar parental attitudes toward their children in relation to PA goals. Lipowska et al. (39) also concluded that the child's gender was not significant in relation to the nature of parental attitudes, but observed that it did matter in the context of sexism. The study by Lipowska et al. (39) on sexism in relation to parental attitudes showed that the mothers with the highest levels of sexism were characterized by inconsistent and demanding attitudes. Overprotectiveness and attitudes of autonomy were ranked the lowest in terms of their relationship with sexism. Mothers exhibit higher levels of acceptance–rejection, demanding attitudes, and attitudes of autonomy than do fathers. Education levels also played a role in this study: a higher level of education in the mother was linked to high levels of demandingness and low levels of autonomy.

Fathers with vocational education exhibited stronger attitudes of autonomy toward their offspring. With regard to the parents of preschoolers surveyed previously, the role of sexism appeared to be most notable in the attitudes of mothers toward their sons (39). In the authors' own research, the most important attitudes identified in mothers were acceptance–rejection, overprotectiveness, demandingness, and attitudes of autonomy. The attitudes of autonomy and acceptance–rejection were also significant in fathers. Similar conclusions were obtained here is in previous studies on sexism by Lipowska et al. (39). In both studies, significant relationships were identified with mothers' attitudes of autonomy, demandingness, and overprotectiveness.

Lipowski et al. (17) examined two groups of parents: former and current athletes. Among the families surveyed, 152 mothers were former athletes, and only 61% of them continued to play sports regularly; 198 fathers were former athletes, of whom 171 (86%) continued to play sports regularly. Although Lipowski et al. (17) did not directly report on parental attitudes, they touched on an important aspect of PA in the family in relation to parents' feeding styles. The authors observed significant differences between the mothers who currently practiced sports and those who did not practice sports regularly: mothers who engaged in sporting activities (either organized or on their own) used a controlling and encouraging parenting style more often and displayed a lesser tendency toward the emotional feeding style compared to women who did not exercise. In addition, their children spent more hours per week on sports. A similar pattern was observed in the comparison between fathers who were regularly exercising and those who were not doing so. Less frequent use of control and incentive-based parenting styles was observed in families in which neither the mother nor the father practiced sports regularly. In families where both parents regularly engaged in sporting activities, parents more frequently engaged in an emotional style of eating compared to families where only one parent practiced sports or neither of the parents did (17). Comparing the results obtained by Lipowski et al. (17) with the results of this study, it can be observed that PA among mothers and fathers affects both their eating styles and their parental attitudes. Less involvement in sports results in a lower degree of control in eating styles among both mothers and fathers. A similar relationship was observed in the present study between the goals of PA undertaken by parents and their parental attitudes. The more acceptance shown by a mother toward her child, the lower the strength of her need to achieve the goal of boosting confidence.

Additionally, a strong attitude of acceptance toward the child was found to increase the strength of the goal of well-being. Stronger goals of attaining a shapely body and managing stress increased mothers' attitudes of autonomy toward their offspring. It can be concluded that mothers who are less controlling with respect to their own goals are more accepting of autonomy in their offspring. For fathers, a stronger goal of boosting confidence was linked to greater autonomy toward the child. It can also be concluded that such fathers may have less need to control their offspring's engagement in PA during their leisure time.

Parental attitudes are rarely analyzed in the context of children's involvement in PA. Cheung (45) examined the attitude of parents toward out-of-school PA among children attending school (early school education). The parents agreed that the children did not get enough PA outside school, mentioning the sheer volume of activities that occur outside school, such as doing homework and watching TV. Cheung confirmed that parents do not always facilitate PA during leisure time, but argued that this is crucial for their children (45).

In their own research, the authors have found that parental attitudes are, to a small extent, responsible for children's participation in PA, or lack thereof, in their leisure time during middle childhood. Their own PA goals also play a major role. Tandon et al. (25) confirmed that parents and caregivers believe that daily PA is essential for preschoolers, but caregivers of children rate the importance of spending time outdoors during the day much more highly than do parents.

Leung et al. (46) examined parents and children aged 6–9 years in the context of support provided for PA (measured by the Parent Support Scale) and found that parental support had a direct impact on children's PA.

In the authors' own research, attitudes of autonomy among fathers and mothers were found to influence their PA goals. A mother's attitude of autonomy toward her offspring influenced the child's non-PA leisure time, and the strength of a mother's demanding attitude influenced her assessment of the child's fitness. In fathers, the strength of inconsistency in attitude influenced his assessment of the child's fitness and the child's non-PA leisure time. Supporting and encouraging children to engage in PA is important to parents, and together with attitudes and parenting styles, this support and encouragement can affect children's engagement in PA during their leisure time in middle childhood.

Our own research and that of Leung et al. (46), Cheung (45), and Tandon et al. (25) has demonstrated that parents have a significant influence on PA among their children in the period of middle childhood and the early school years. Alpgan et al. (42) investigated parental attitudes and the opinions of parents regarding the readiness of children (5–6 years). They reported that Metropolitan Readiness Test (MRT) scores are significantly negatively correlated with scores on the subscales of the Parental Attitude Research Instrument (PARI) measuring overprotectiveness, refusal to be a housewife, husband–wife conflict, and strict discipline. Specifically, as children's MRT scores increase, there is a decline in parental overprotectiveness, refusal to be a housewife, husband–wife conflict, and strict discipline. The mean scores for refusal to be a housewife, husband–wife conflict, and strict discipline were higher for parents of children who were not ready for school than for those who were ready for school. Negative attitudes within the family reduce children's readiness for school (47).

Similar conclusions can be drawn from our own research: inconsistent attitudes in fathers reduced their assessment of their children's fitness and were linked to increased non-PA leisure time. However, mothers' attitudes of autonomy were also associated with an increase in children's non-PA leisure time. An excessively demanding attitude contributed to a better assessment of the child's fitness by the mother. Parents' attitudes toward their own children exert indirect effects on both readiness to start learning and engagement in PA. Lindsay et al. (48) identified health-related parenting practices related to PA, which included PA modeling, engaging in PA with the child, and encouraging the child to engage in PA. Mothers emphasized the importance of parents as role models and exerted a positive effect on children's PA. In addition, the authors identified parenting practices that limit children's PA (48).

Our research shows that parental attitudes depend on parents' PA-related goals, and it is these goals that in turn affect the engagement of children in PA during their leisure time. Comparing this research to that of Lindsay et al. (48) confirms that parents' behaviors and practices influence how their preschool-aged children spend their time. The abovementioned studies were qualitative in nature and were conducted in a similar age group. However, this research was transverse in design and used standardized questionnaires and scales to assess the goals of PA and parental attitudes. In the future, this study may form the basis for the initiation of longitudinal studies and the observation of children not only at preschool age but also at school age. This area of research may offer the possibility for transgenerational observation of children and their families from the point of view of PA within the family.

## 5. Limitations

The study was cross-sectional and observational in nature. The study employed not only questionnaires and scales to objectively assess parents' attitudes toward their offspring but also parents' subjective assessments of their children's PA. Parents provided information about their offspring's PA, which might have resulted in less objective assessments of their children, but the authors used a 5-point scale to reduce parental subjectivity in assessing their child's PA.

No randomization or blinding of the study group was used in this study; this was due to the lack of a control group. The aim of the study was not to compare parental attitudes toward children across different age groups. Instead, the authors collected data on a large group of parent–child dyads to investigate the relationships between parental attitudes, parental PA-related goals, and children's PA during their leisure time. The lack of longitudinal analysis was also related to the deliberate selection of the age group of the children; no attention was paid to the observation of changes in parental attitudes toward their offspring over a specific time frame. However, it would be worthwhile to carry out a longitudinal study in the future and compare the parental attitudes of children in middle and late childhood, investigating how these attitudes affect PA during leisure time. Longitudinal surveys require that participants be sufficiently motivated to complete the survey procedure. The transverse nature of the present study was associated with the desire to test a large group of parent–child dyads and obtain the most accurate results subject to statistical analysis.

## 6. Conclusion

The results indicated that not all parental attitudes had a direct impact on children's PA and non-PA leisure time. A mother's attitude of autonomy toward her children had a direct impact, reducing children's non-PA leisure time. Among fathers, it transpired that an inconsistent attitude was significant is increasing the child's non-PA leisure time. Other aspects of parental attitude did not have a significant effect on children's leisure time. However, the goals of parents' PA were found to influence children's PA and non-PA leisure time. In mothers, goals such as being active, being fit, and promoting PA, for example, had a direct impact on whether their children were active or not during their leisure time. Fathers' PA goals also directly influenced the quality of their offspring's leisure time. The parental PA-related goal that most strongly influenced PA and non-PA leisure time among their children was the desire for a fit, shapely body. The goals of fathers differed from those of mothers in this study. The most statistically significant relationships for both mothers and fathers were between parental attitudes and PA goals. The results of this study suggest that parental attitudes do not play a significant role in explaining engagement in PA, or lack thereof, during leisure time among 5-year-old children.

## Data availability statement

The raw data supporting the conclusions of this article will be made available by the authors, without undue reservation.

## Ethics statement

The studies involving human participants were reviewed and approved by the Ethics Board for Research Projects at the Institute of Psychology, University of Gdansk, Poland (Decision No. 17/2013). Written informed consent to participate in this study was provided by the participants' legal guardian/next of kin.

## Author contributions

Conceptualization, methodology, investigation, data curation, supervision, and funding acquisition: MLipowska and MLipowski. Software: MLipowski and SL. Writing—original draft: AK, MLipowska, and SL. Writing—review and editing: AK, MLipowska, and SL. Project administration: MLipowska. All authors have read and agreed to the published version of the manuscript.
